# Supplementary figures and images for: CFAP53 regulates mammalian cilia-type motility patterns through differential localization and recruitment of axonemal dynein components
Source: PLoS Genet. 2020 Dec 21;16(12):e1009232. doi: 10.1371/journal.pgen.1009232 (PMC7817014; doi:10.1371/journal.pgen.1009232)

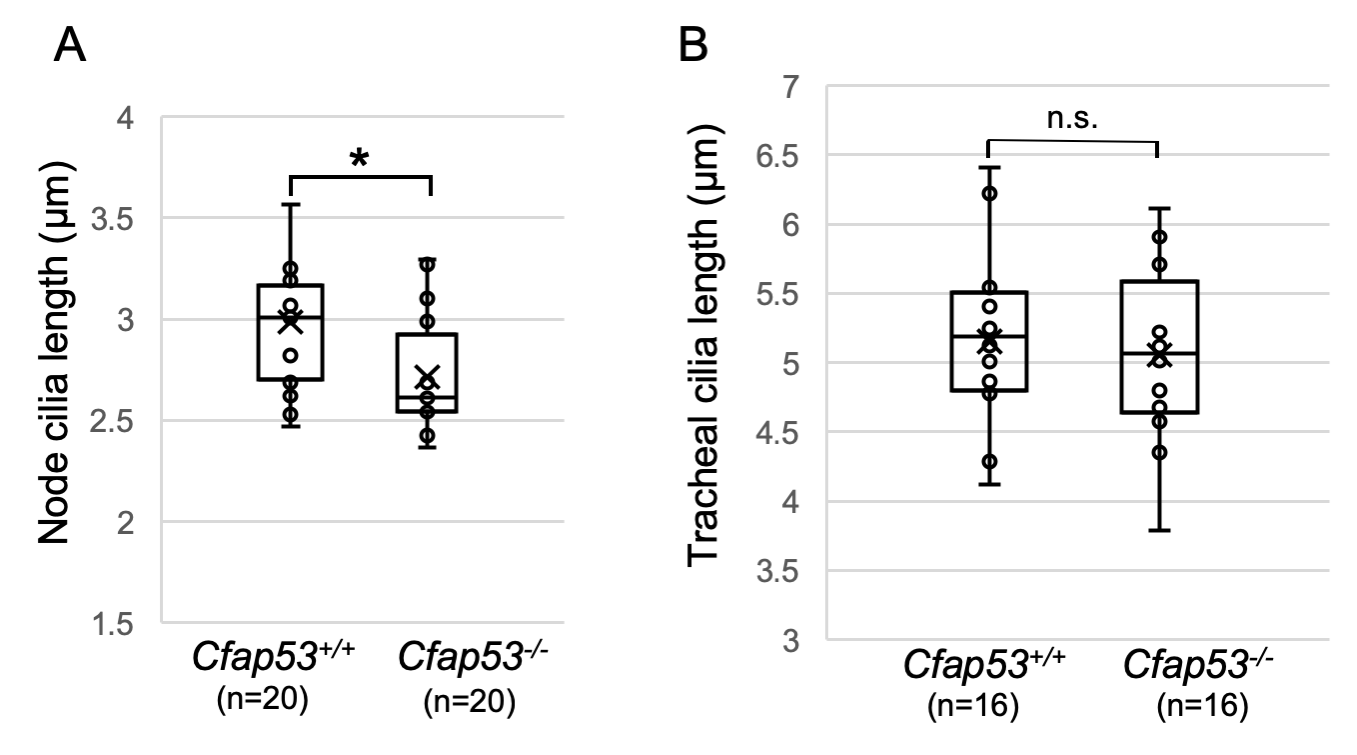

Supplement: S1 Fig — (A) The length of node cilia from Cfap53+/+ and Cfap53-/- embryos. Data are presented as mean ± SD (n = 20 independent variables); two tailed Student’s t-test (*p = 0.0109). (B) The length of tracheal cilia from Cfap53+/+ and Cfap53-/- mice. Data are presented as mean ± SD (n = 16 independent variables); two tailed Student’s t-test (n.s.; p = 0.6608). Note that mutant node cilia are slightly shorter but mutant tracheal cilia retained the normal length. (TIF) [file pgen.1009232.s001.tif]

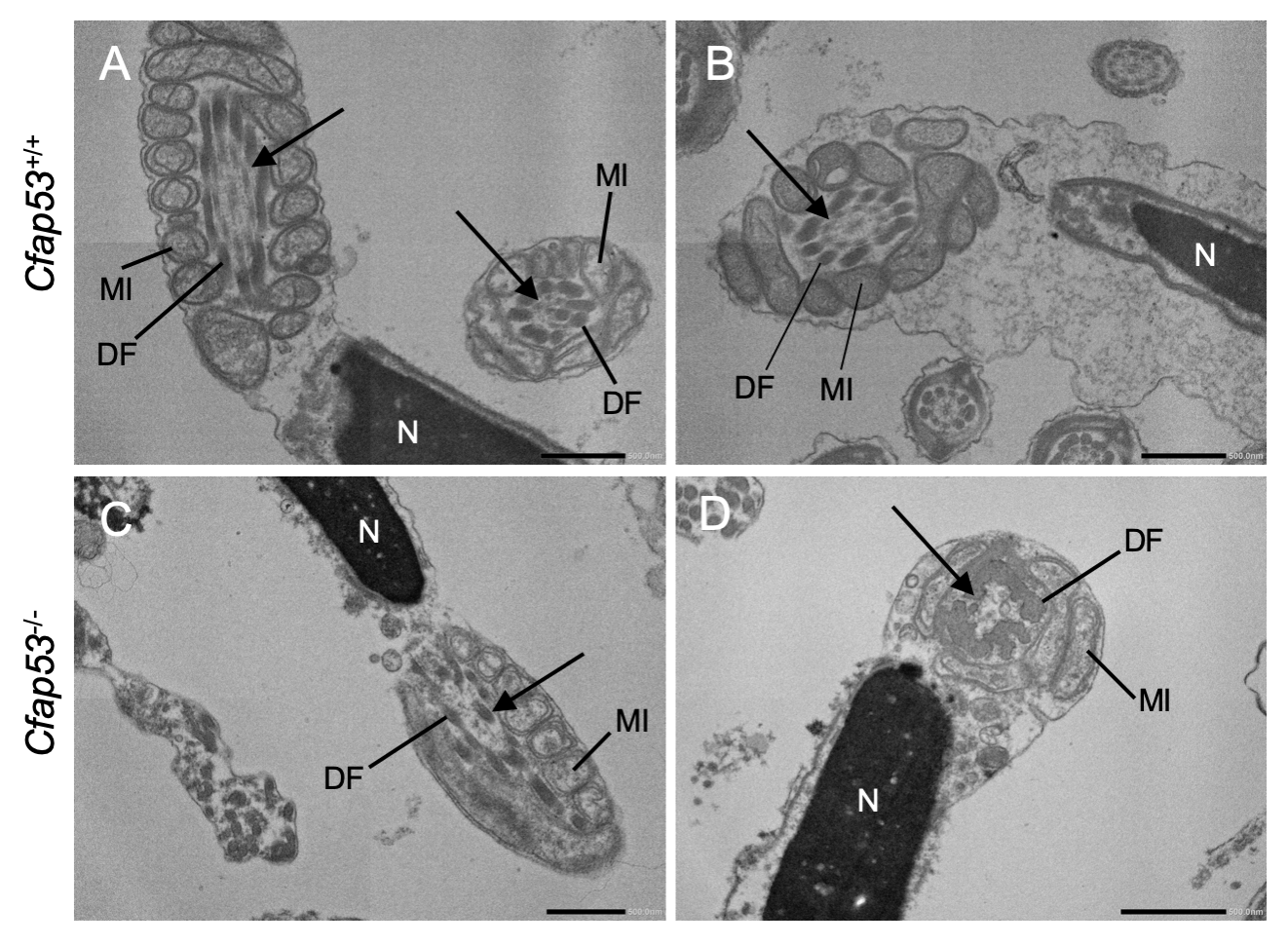

Supplement: S2 Fig — Sperm from Cfap53+/+ and Cfap53-/- male mice was subjected to TEM analysis. Note that normal axonemal structure is severely disrupted in the mutant sperm. Arrows indicate the center of the cross section of sperm. Note that the 9+2 structure is observed in Cfap53+/+, but is disrupted in Cfap53-/-. N: Nucleus, DF: Outer dense fiber, MI: Mitochondrion. Scale bars, 500 nm. (TIF) [file pgen.1009232.s002.tif]

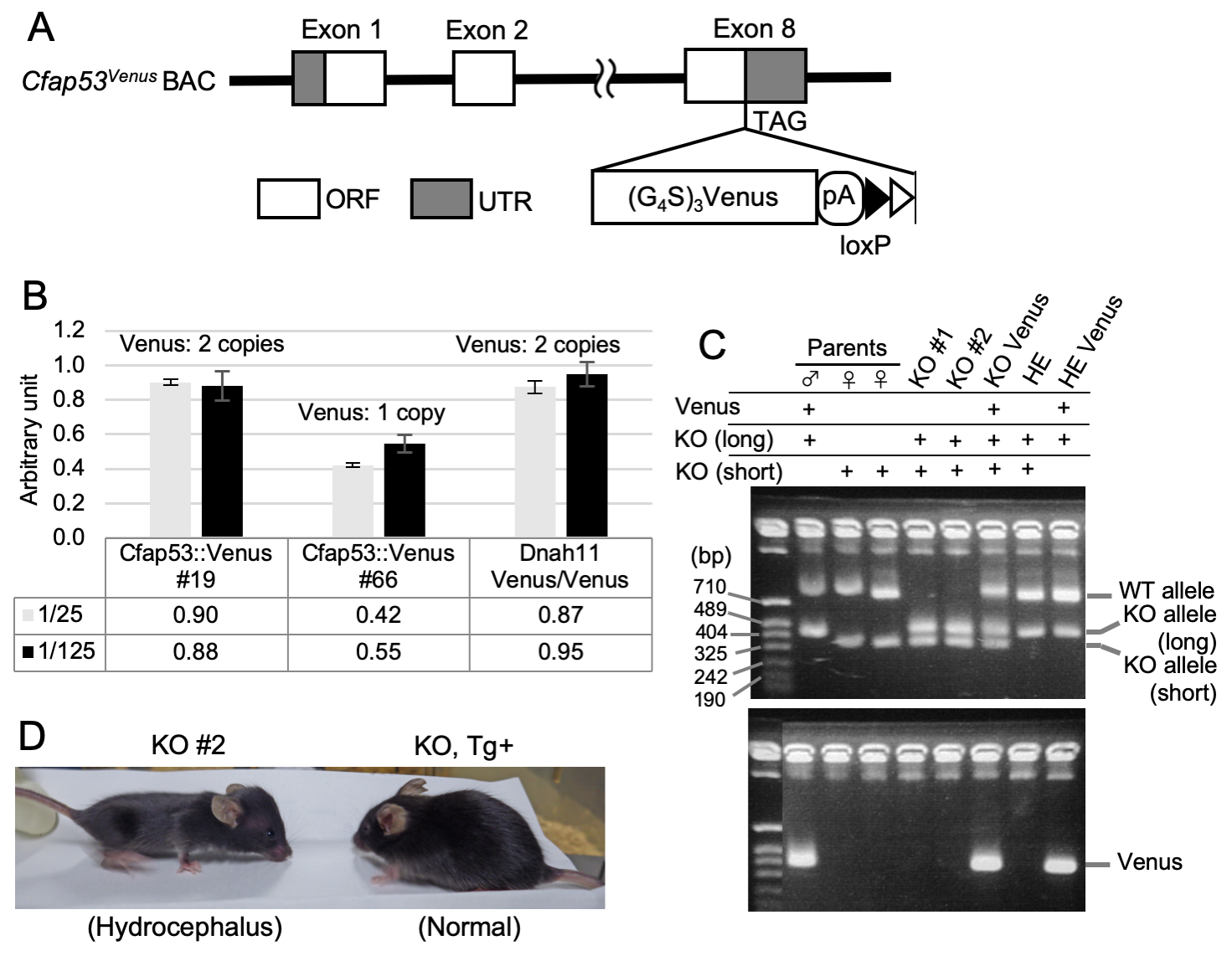

Supplement: S3 Fig — (A) Construction of a transgenic mouse strain harboring a Cfap53Venus BAC. The coding sequence for Venus together with that for a (Gly4Ser)3 linker and poly(A) (pA) sequence as well as a loxP site was inserted into exon 8 of Cfap53 gene immediately upstream of the TAG stop codon. ORF, open reading frame; UTR, untranslated region. (B) Copy number of the BAC transgene was examined for two transgenic lines (#19, #66) harboring the Cfap53Venus BAC by quantitative PCR. Genomic DNA from Dnah11Venus/Venus mouse, which has two copies of Venus, served as a control. Note that lines #19 and #66 have 2 copies and 1 copy of the transgene, respectively. (C) Genotyping of Cfap53-/- with Cfap53Venus. Cfap53-/-, exon 2 excluded, have two alleles of different length. The male, Cfap53+/- (long alleles: used in other experiments) with Cfap53Venus, was crossed with females, Cfap53+/- (short alleles). We can distinguish between Cfap53-/-, Cfap53Venus and Cfap53+/-, Cfap53Venus by length of Cfap53-/- alleles in F1 mice. (D) Mice genotyped in (C) at 4 weeks of age. Hydrocephalus apparent in Cfap53–/–(KO) mice was rescued by introduction of the Cfap53Venus transgene. (TIF) [file pgen.1009232.s003.tif]

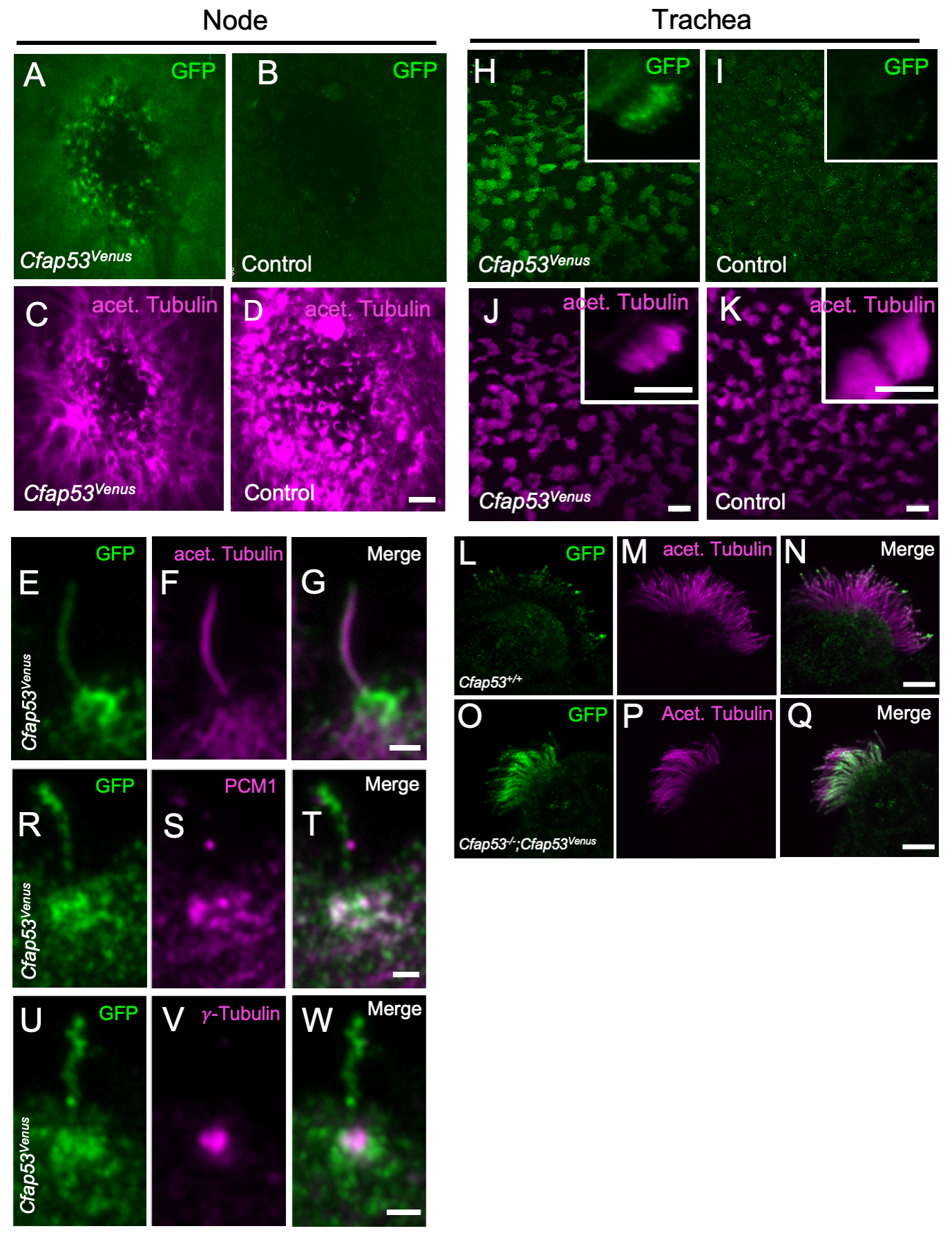

Supplement: S4 Fig — (A–D) Immunofluorescence staining of the node of Cfap53Venus (A and C) or control (B and D) embryos at E8.0 with antibodies to GFP (A and B) and to acetylated (acet.) Tubulin (C and D). Scale bar, 10 μm. (E–G) Higher magnification images of a node cilium of a Cfap53Venus embryo showing GFP, acetylated Tubulin, and merged staining, respectively. CFAP53::Venus was predominantly localized at the base of node cilia but was also detected at a much lower level in the axoneme. Scale bar, 1 μm. (H–K) Immunofluorescence staining of the trachea of adult Cfap53Venus and control mice with antibodies to GFP (H and I) and to acetylated (acet.) Tubulin (J and K). Scale bars, 20 μm. Insets in H, I show higher magnification views of tracheal cilia. Scale bars, 5 μm. (L–Q) Immunofluorescence analysis of an isolated tracheal MCC from a Cfap53-/- mouse harboring the Cfap53Venus transgene (O-Q) or a Cfap53+/+ mouse without the transgene (L-N). CFAP53::Venus was absent in the distal region of tracheal cilia (O-Q). Scale bars, 5 μm. (R-T) Immunofluorescence staining of the node cilia of Cfap53Venus embryos at E8.0 with antibodies to GFP (R) and to PCM1 (S). Scale bar, 1 μm. (U-W) Immunofluorescence staining of the node cilia of Cfap53Venus embryos at E8.0 with antibodies to GFP (U) and to γ-Tubulin (V). Scale bars, 1 μm. (TIF) [file pgen.1009232.s004.tif]

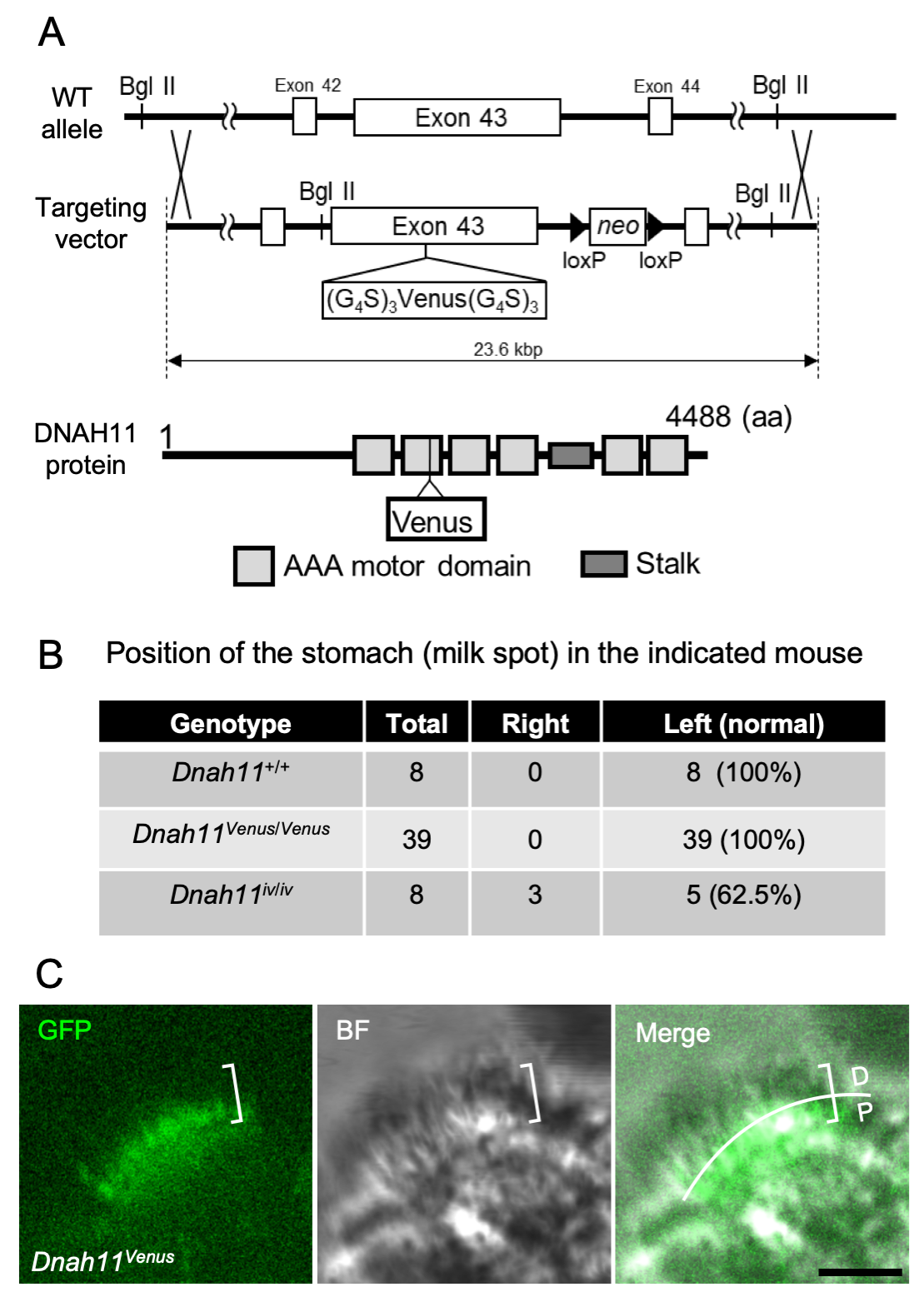

Supplement: S5 Fig — (A) Construction of a transgenic mouse strain harboring a Dnah11Venus allele. The targeting vector contained a neo cassette and loxP sites, and the coding sequence for Venus was inserted into exon 43 (which encodes the second AAA motor domain) of Dnah11 together with that for (Gly4Ser)3 linkers. (B) Laterality of milk spots for WT, Dnah11Venus/Venus, and Dnah11iv/iv (homozygous for a spontaneous Dnah11 mutation that results in situs inversus). All milk spots of Dnah11Venus/Venus mice were located on the left (normal) side (n = 39/39), whereas those of Dnah11iv/iv mice were L-R randomized (n = 3/8). (C) Live fluorescence imaging of DNAH11::Venus (green) in adult tracheal cilia of mice harboring a Dnah11Venus. DNAH11::Venus was detected in proximal region of tracheal cilia. BF, bright-field. D and P in the merged image indicate the distal and proximal region of cilia, respectively. Scale bar, 5 μm. (TIF) [file pgen.1009232.s005.tif]

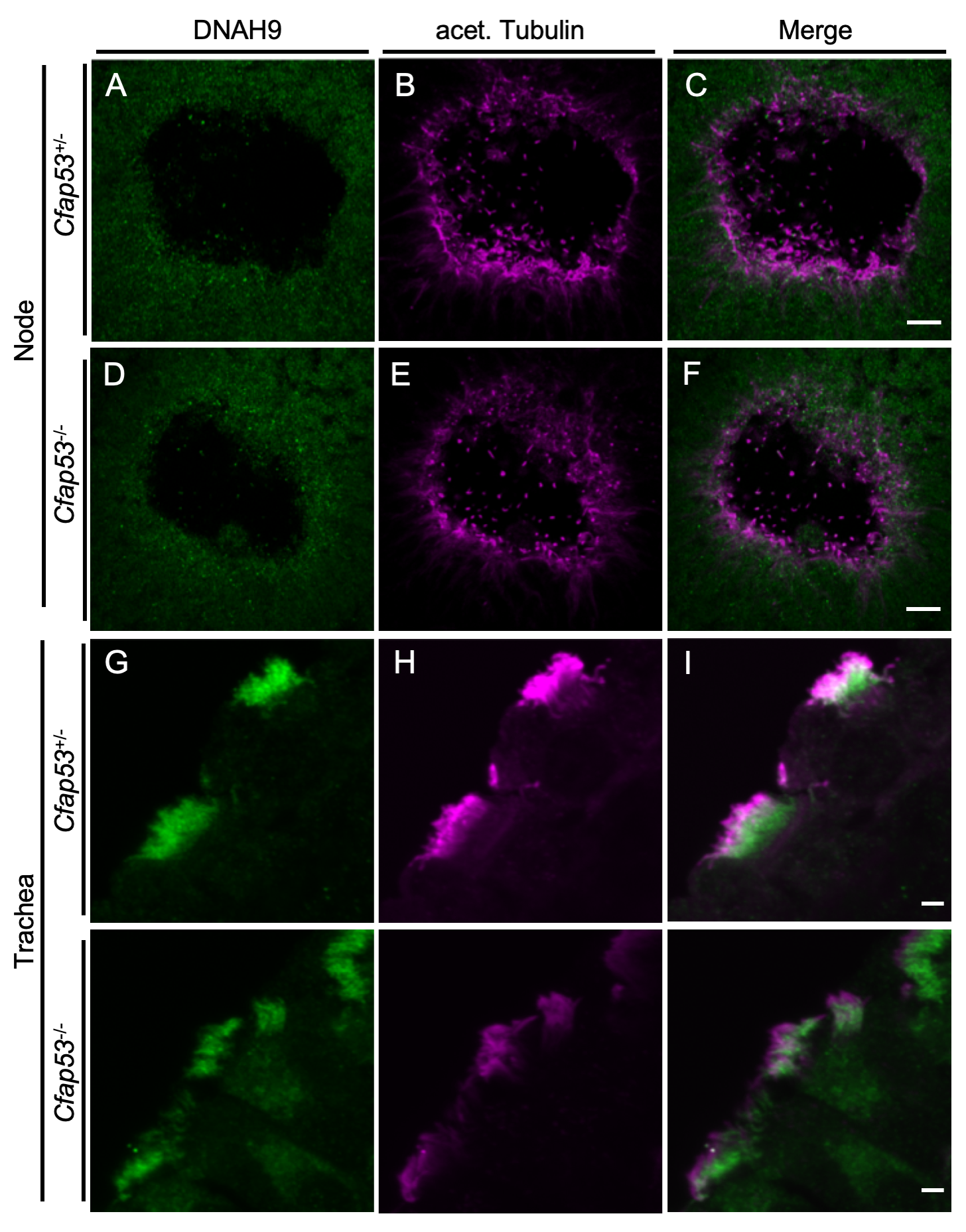

Supplement: S6 Fig — (A-F) Immunofluorescence staining with antibodies to DNAH9 (green) and to acetylated (acet.) Tubulin (magenta) of the node at E8.0 and adult trachea (G-L) of Cfap53–/–and control mice. DNAH9 was not detected in node cilia of Cfap53+/–or Cfap53–/–embryos (A-F), whereas its expression was maintained in tracheal cilia of the mutant (G-L). Scale bars, 10 μm (A-F) or 2 μm (G-L). (TIF) [file pgen.1009232.s006.tif]

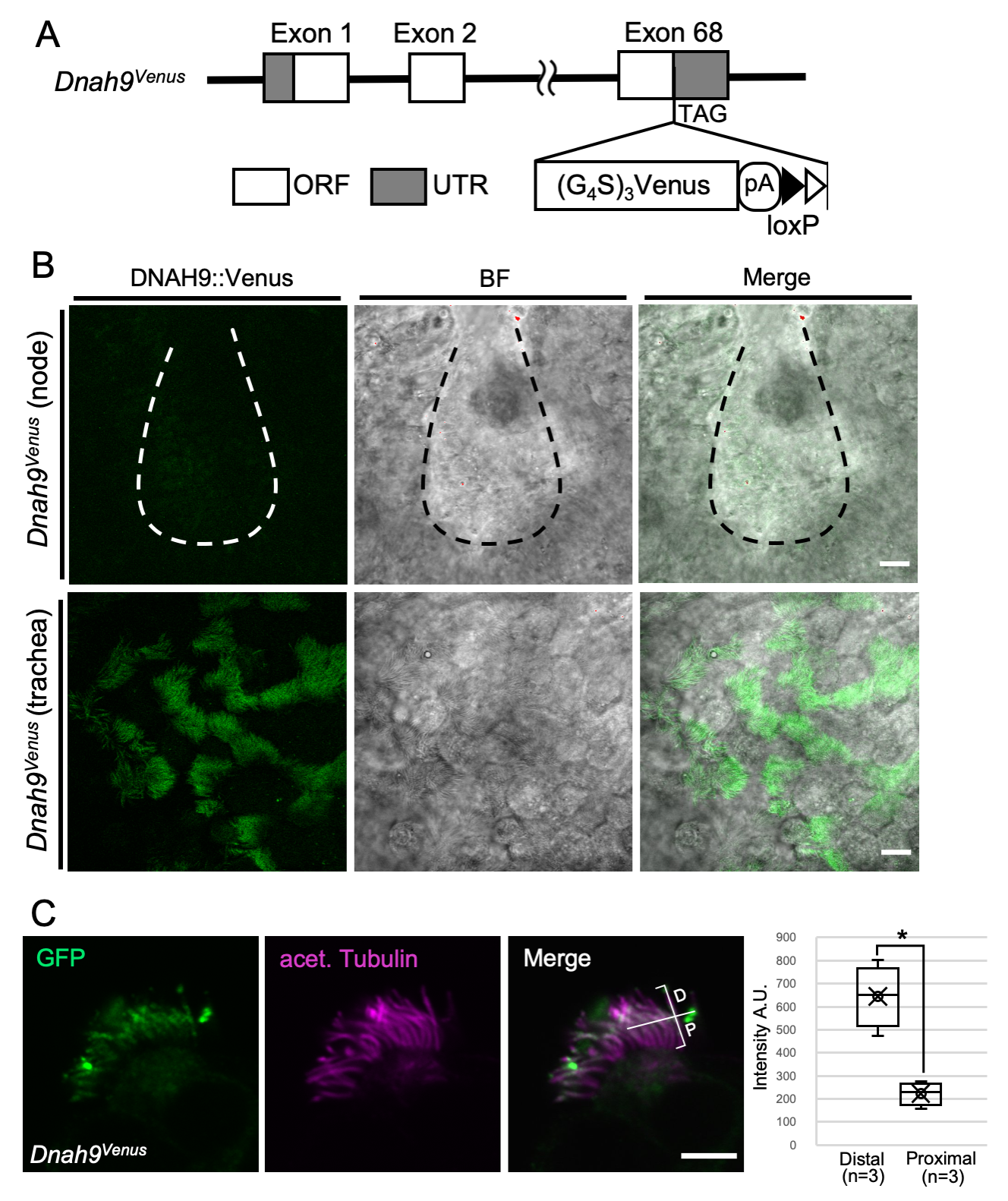

Supplement: S7 Fig — (A) Dnah9Venus allele was generated by inserting (G4S)3 Venus at the carboxyl terminus of DNAH9. (B) Live fluorescence imaging of DNAH9::Venus (green) in the node at E8.0 and in adult tracheal cilia of mice harboring a Dnah9Venus transgene. DNAH9::Venus was detected in tracheal cilia but not in node cilia. The dashed lines indicate the outline of the node. Bright-field (BF) and merged images are also shown. Scale bars, 10 μm. (C) Immunofluorescence staining with antibodies to GFP (green) and to acetylated (acet.) Tubulin (magenta) of adult trachea from Dnah9Venus mice. Note that DNAH9::Venus is preferentially localized to the distal region of tracheal cilia. D and P indicate and distal and proximal regions of tracheal cilia, respectively. Quantitative analysis confirms a higher intensity of GFP signals in the distal region. Data are presented as mean ± SD (n = 3 independent variables); two tailed Student’s t-test (*p = 0.0144). Scale bar, 5 μm. (TIF) [file pgen.1009232.s007.tif]

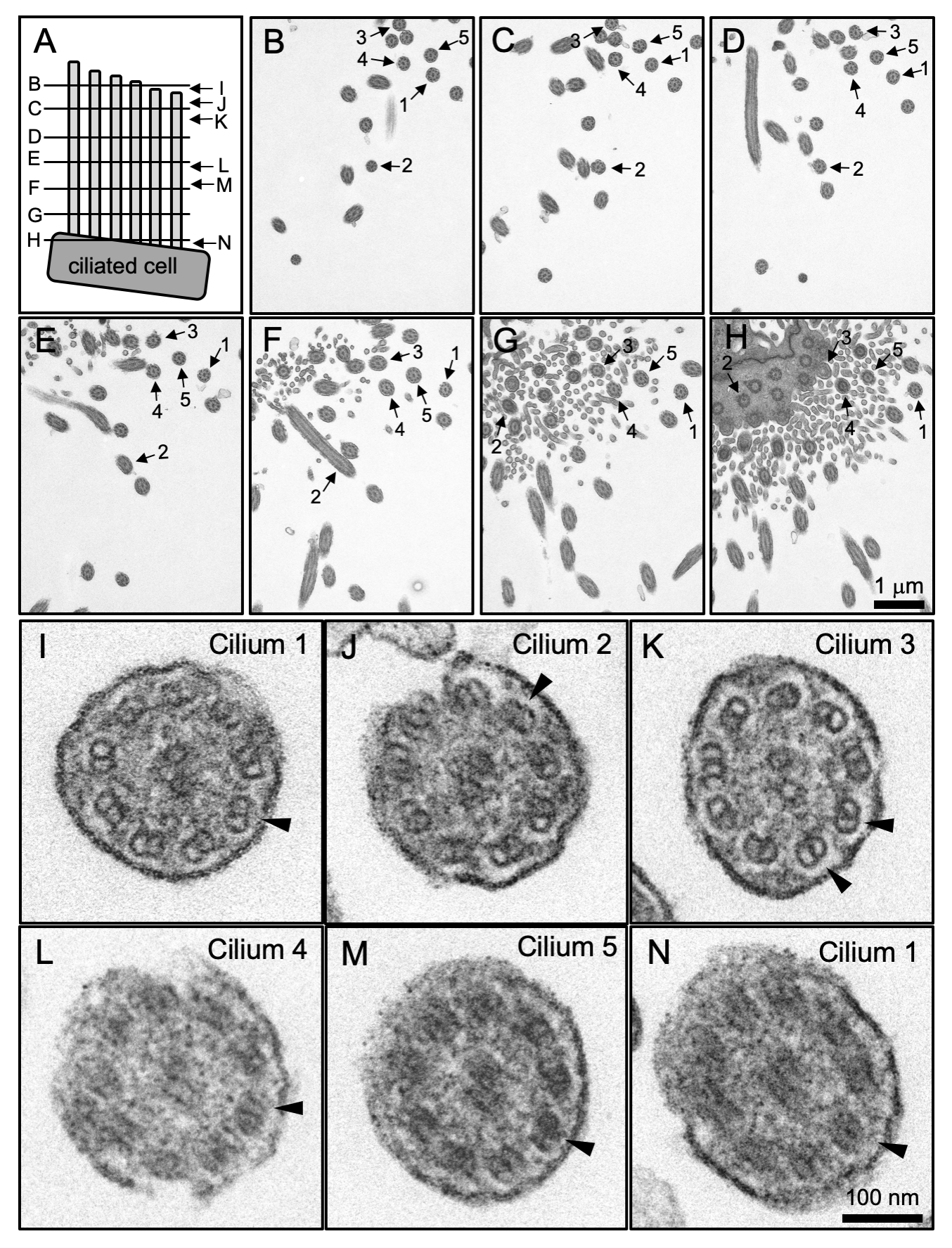

Supplement: S8 Fig — (A) Serial TEM sections were made from Cfap53-/- tracheal cilia along the proximal-distal axis. The level of each section shown in (B-N) is illustrated. (B-H) Lower magnification images of seven sections. Scale bar, 1 μm. (I-N) Higher magnification images. Scale bar, 100 nm. The cilium from which the high magnification image is derived is indicated at the right-top of each panel. For example, panel (I) shows a section image of cilium 1 at the level indicated in (A). Arrowheads denote microtubules lacking ODA. (TIF) [file pgen.1009232.s008.tif]

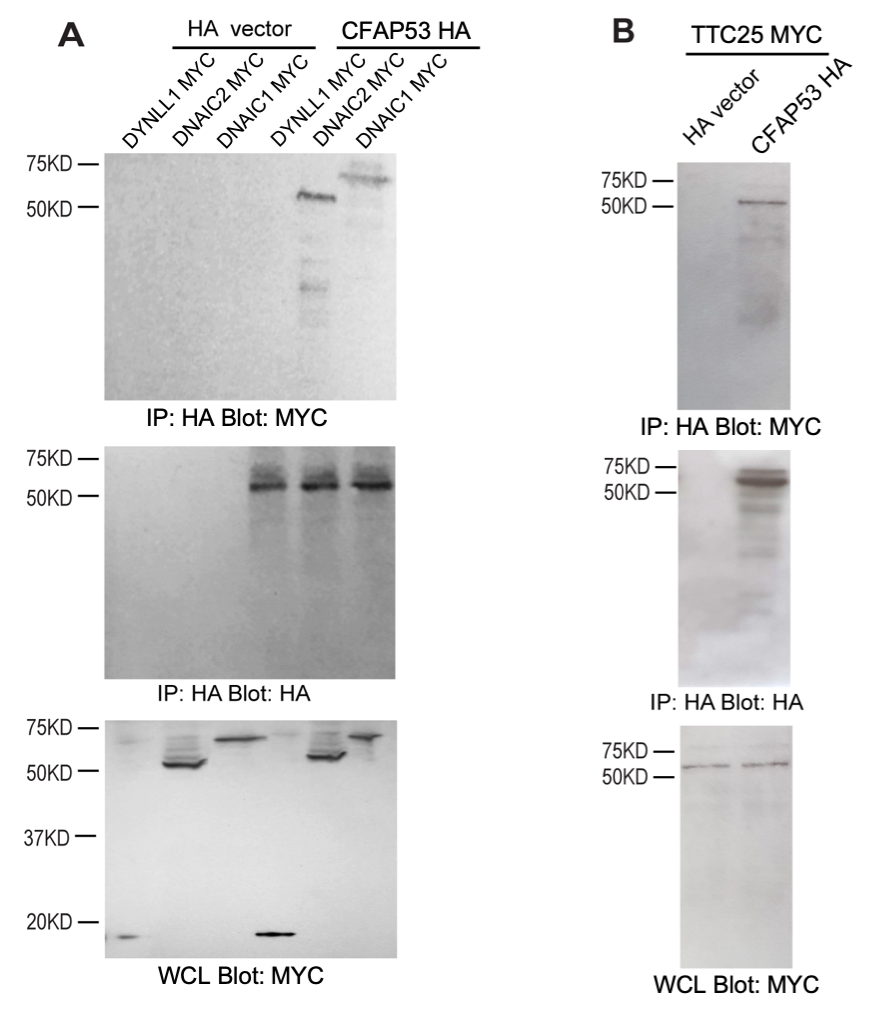

Supplement: S9 Fig — (A-B) HEK293T cells transfected with expression vectors for HA-tagged CFAP53 or MYC epitope–tagged DNAIC1, DNAIC2, DYNLL1 (cytoplasmic dynein light chain), TTC25 (or with the corresponding empty vectors), as indicated, were subjected to immunoprecipitation (IP) with antibodies to HA or to MYC, and the resulting precipitates as well as the original whole cell lysates (WCLs) were subjected to immunoblot analysis with antibodies to HA or MYC. CFAP53 interacted specifically with the axonemal dyneins DNAIC1, DNAIC2 and the DC member TTC25. (TIF) [file pgen.1009232.s009.tif]

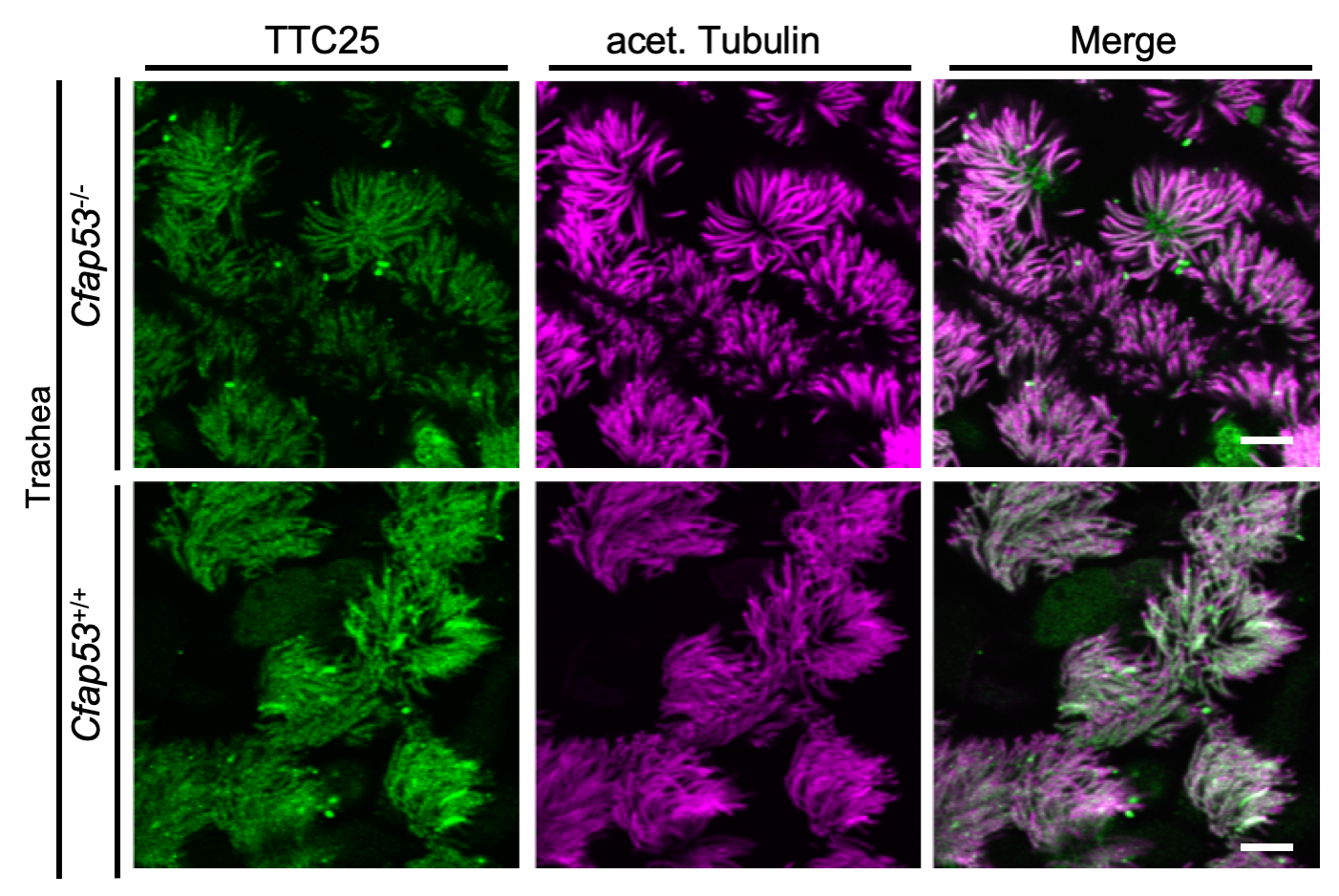

Supplement: S10 Fig — Tracheal cilia of adult Cfap53–/–and control mice were subjected to immunofluorescence staining with antibodies to TTC25 (green) and to acetylated (acet.) Tubulin (magenta). TTC25 was maintained in tracheal cilia of Cfap53–/–mice, albeit at a reduced level. Scale bars, 5 μm. (TIF) [file pgen.1009232.s010.tif]

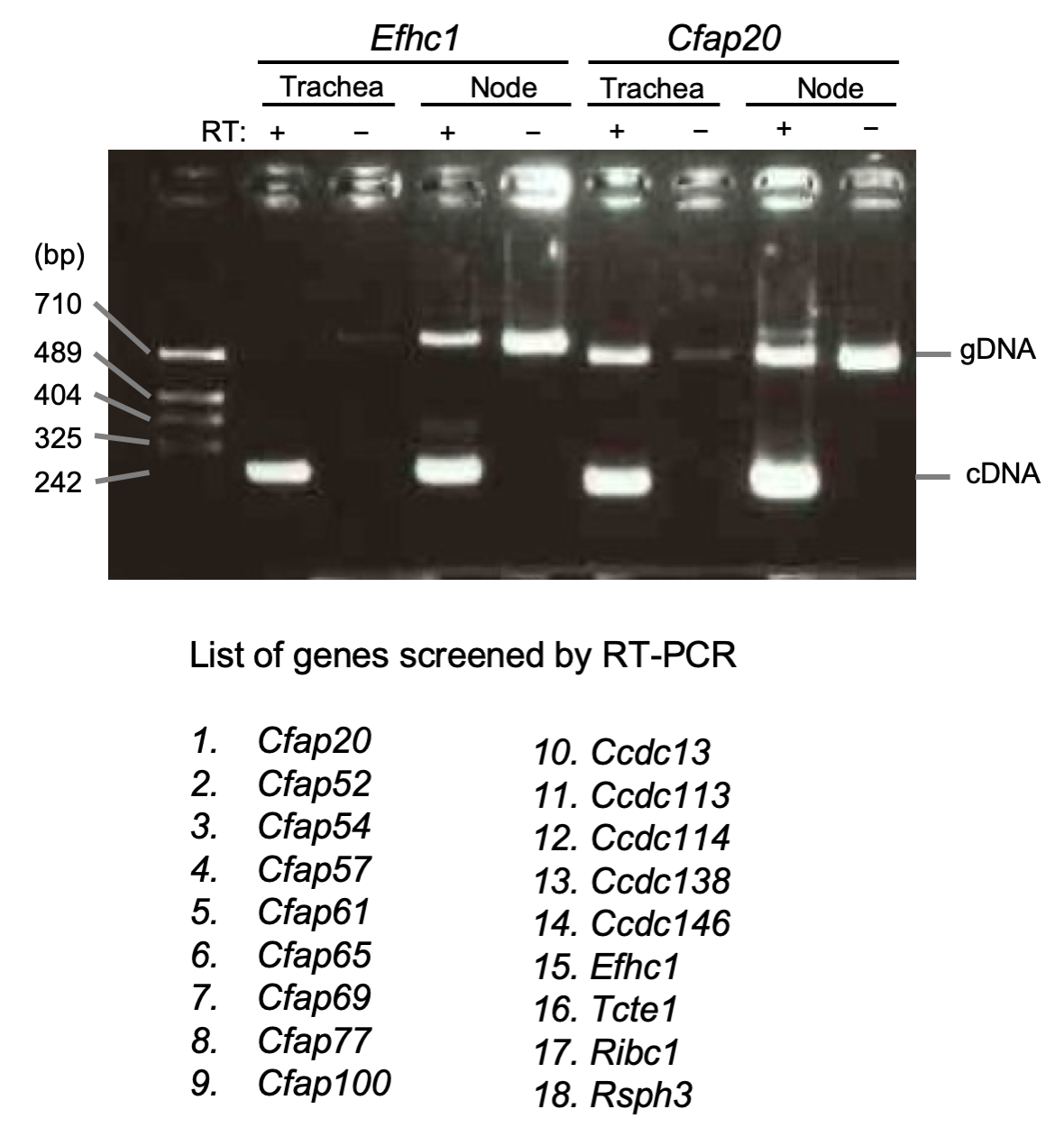

Supplement: S11 Fig — Total RNA isolated from adult trachea or E8.0 node of mice was subjected to RT-PCR analysis of the 18 indicated genes. Amplified cDNAs were detected by agarose gel electrophoresis. Representative results for Efhc1 and Cfap20 are shown. No gene was found to be specifically expressed in tracheal ciliated cells and not in node ciliated cells. (TIF) [file pgen.1009232.s011.tif]
