# Supplementary material for: CFAP53 regulates mammalian cilia-type motility patterns through differential localization and recruitment of axonemal dynein components
Source: PLoS Genet. 2020 Dec 21;16(12):e1009232. doi: 10.1371/journal.pgen.1009232 (PMC7817014; doi:10.1371/journal.pgen.1009232)
Supplement: Attachment — Submitted filename: PGenetics-D-20-00551.pdf [file pgen.1009232.s024.pdf]

Differential requirement of Cfap53 for ciliary motility in 9+0 and 9+2 motile cilia

The manuscript by Ide et al. is a thorough investigation of the role of Cfap53/CCDC11 in the motility of nodal (9+0) and tracheal (9+2) cilia in the mouse. It is based on earlier work in humans, frogs, and zebrafish that indicated a requirement for Cfap53/CCDC11 in the determination of laterality. However, this study extends prior work by providing a more detailed analysis of the localization of Cfap53/CCDC11 in different tissues and by assessing the impact of null mutations on ciliary motility and the assembly of outer dynein arm subunits and docking proteins.

Cfap53-LacZ constructs indicate that expression of Cfap53 is limited to pit cells of the embryonic node (which assemble motile nodal cilia) and other embryonic and adult tissues that assemble motile cilia (Figure 1). Knockout of the Cfap53 gene results in defects in laterality such as situs inversus and heterotaxy and hydrocephalus of the brain. Detailed analysis of ciliary motility shows that nodal cilia are immotile, whereas the motility of cilia in the trachea and brain ventricles is abnormal (Figure 2). Transformation with a CFAP53::Venus construct rescues the mutant defects. Immunofluorescence reveals that the Cfap53 fusion protein is localized near the base of the nodal cilia and pericentriolar satellite region. The protein is also seen in the proximal region of the axoneme in tracheal cilia (Figure 3).

To better understand how loss of Cfap53 affects the motility of cilia, the authors also analyzed the distribution of three dynein heavy chains, Dhc5, Dhc11, and Dhc9, in nodal and tracheal cilia. Dhc5 was found in WT cilia, missing in nodal cilia from the null mutant, and present but reduced in tracheal cilia of the mutant (Figure 4). Dhc11 was also missing in nodal cilia of the mutant but present in the proximal region of tracheal cilia at reduced levels in the mutant (Figure 5). The results with Dhc9 were somewhat surprising; it was not detected in either WT or mutant nodal cilia, in contrast to previous work on human cilia (Figure 6). Dhc9 was localized to the distal region of tracheal cilia but in reduced amounts in mutant cilia. Additional studies by transmission electron microscopy confirmed that the outer dynein arms were missing in nodal cilia and reduced in tracheal cilia (Figure 7). Loss appeared to biased to specific outer doublets (#3 and #8) that could be correlated with the direction of planar beating. Analysis of double mutants also lacking the radial spokes enhanced the defects in the assembly of the outer dynein arms.

To determine if Cfap53 interacted directly with outer arm dynein subunits or docking proteins, the authors performed selective extraction and co-immunoprecipitation experiments (Figure 8). Cfap53 was not extracted by 0.6M NaCl, but it was released by 0.6M NaI extraction. Co-IP experiments suggested a possible interaction between Cfap53 and dynein subunits and one docking complex protein, Ttc25. To further investigate the potential interaction between Cfap53 and Ttc25, the authors performed immunolocalization studies in different mutant backgrounds (Figure 9). Cfap53 was still detected in nodal cilia of the *Ttc25-/-* mutant, suggesting the Cfap53 does not depend on Ttc25 for ciliary localization. However, Ttc25 was missing in nodal cilia and reduced in tracheal cilia from the *Cfap53-/-* mutant, consistent with a requirement for CFAP53 in the localization of outer arm docking proteins and subunits, at least in nodal cilia.

The above studies, especially the localization of proteins in the embryonic node, are technically challenging and nicely executed. The work is summarized diagrammatically in Figure 10. <u>My only reservation is the final diagram (Figure 10b) suggesting a potential role for Cfap53 in the interaction between the outer dynein arm subunits and docking proteins and the IFT machinery.</u> None of the experiments in the present study provide any evidence for an interaction of the Cfap53 protein with the IFT machinery. The authors should either clarify their diagram and/or discuss what other evidence they have to support this model. Given recent work identifying CFAP53 as a MIP subunit in Chlamydomonas flagella, it would also seem prudent to consider other models for its function in mouse cilia.
