# Supplementary material for: CFAP53 regulates mammalian cilia-type motility patterns through differential localization and recruitment of axonemal dynein components
Source: PLoS Genet. 2020 Dec 21;16(12):e1009232. doi: 10.1371/journal.pgen.1009232 (PMC7817014; doi:10.1371/journal.pgen.1009232)
Supplement: Attachment — Submitted filename: Response to reviewers.docx [file pgen.1009232.s025.docx]

**Response to the reviewers’ comments**

**Reviewer #1:**

*The manuscript by Ide et al. is a thorough investigation of the role of Cfap53/CCDC11 in the motility of nodal* *(9+0) and tracheal (9+2) cilia in the mouse. It is based on earlier work in humans, frogs, and zebrafish that* *indicated a requirement for Cfap53/CCDC11 in the determination of laterality. However, this study extends* *prior work by providing a more detailed analysis of the localization of Cfap53/CCDC11 in different tissues* *and by assessing the impact of null mutations on ciliary motility and the assembly of outer dynein arm* *subunits and docking proteins.* *Cfap53-LacZ constructs indicate that expression of Cfap53 is limited to pit cells of the embryonic node (which* *assemble motile nodal cilia) and other embryonic and adult tissues that assemble motile cilia (Figure 1).* *Knockout of the Cfap53 gene results in defects in laterality such as situs inversus and heterotaxy and* *hydrocephalus of the brain. Detailed analysis of ciliary motility shows that nodal cilia are immotile, whereas* *the motility of cilia in the trachea and brain ventricles is abnormal (Figure 2). Transformation with a* *CFAP53::Venus construct rescues the mutant defects. Immunofluorescence reveals that the Cfap53 fusion* *protein is localized near the base of the nodal cilia and pericentriolar satellite region. The protein is alsoseen in the proximal region of the axoneme in tracheal cilia (Figure 3).*

*To better understand how loss of Cfap53 affects the motility of cilia, the authors also analyzed the distribution of three dynein heavy chains, Dhc5, Dhc11, and Dhc9, in nodal and tracheal cilia. Dhc5 was found in WT cilia, missing in nodal cilia from the null mutant, and present but reduced in tracheal cilia of the mutant (Figure 4). Dhc11 was also missing in nodal cilia of the mutant but present in the proximal region of* *tracheal cilia at reduced levels in the mutant (Figure 5). The results with Dhc9 were somewhat surprising; it was not detected in either WT or mutant nodal cilia, in contrast to previous work on human cilia (Figure 6).* *Dhc9 was localized to the distal region of tracheal cilia but in reduced amounts in mutant cilia. Additional* *studies by transmission electron microscopy confirmed that the outer dynein arms were missing in nodalcilia and reduced in tracheal cilia (Figure 7). Loss appeared to biased to specific outer doublets (#3 and #8)* *that could be correlated with the direction of planar beating. Analysis of double mutants also lacking the* *radial spokes enhanced the defects in the assembly of the outer dynein arms.*

*To determine if Cfap53 interacted directly with outer arm dynein subunits or docking proteins, the authors performed selective extraction and co-immunoprecipitation experiments (Figure 8). Cfap53 was not extracted by 0.6M NaCl, but it was released by 0.6M NaI extraction. Co-IP experiments suggested a possible interaction between Cfap53 and dynein subunits and one docking complex protein, Ttc25. To further investigate the potential interaction between Cfap53 and Ttc25, the authors performed immunolocalization studies in different mutant backgrounds (Figure 9). Cfap53 was still detected in nodal cilia of the Ttc25-/- mutant, suggesting the Cfap53 does not depend on Ttc25 for ciliary localization. However, Ttc25 was missing in nodal cilia and reduced in tracheal cilia from the Cfap53-/- mutant, consistent with a requirement for CFAP53 in the localization of outer arm docking proteins and subunits, at least in nodal cilia.*

*The above studies, especially the localization of proteins in the embryonic node, are technically challenging and nicely executed. The work is summarized diagrammatically in Figure 10. My only reservation is the final diagram (Figure 10b) suggesting a potential role for Cfap53 in the interaction between the outer dynein arm subunits and docking proteins and the IFT machinery. None of the experiments in the present study provide any evidence for an interaction of the Cfap53 protein with the IFT machinery. The authors should either clarify their diagram and/or discuss what other evidence they have to support this model. Given recent work identifying CFAP53 as a MIP subunit in Chlamydomonas flagella, it would also seem prudent to consider other models for its function in mouse cilia.*

Response: We had proposed an interaction between Cfap53 and the IFT machinery because Cfap53 interacted with Ttc25 (Fig 8) and Ttc25 is known to interact with IFT A and B components (Zu et al., 2015). In view of the reviewer’s comment, we have improved the final figure (Fig 10B) and removed the connection with IFT from the diagram.

**Reviewer #2:**
*Ide and colleagues characterize Cfap53-/- mice in conjunction with other ciliary mouse models (e.g., Dnah9-/-, Rsph4a-/-, etc.) and propose a mechanism for how Cfap53 can differentially regulate nodal / tracheal / ependymal ciliary beating. A particular strength of the study is the characterization of abnormal ciliary waveforms in these ciliated cell types and quantitative analysis of how outer dynein arms are differentially affected in nodal and tracheal cilia. Overall, this study provides novel and interesting insights that advance understanding of the CFAP53 gene and suggest distinct mechanisms that underlie 9+2 as opposed to 9+0 ciliary beating. This manuscript is suitable for PLoS Genetics as the authors use a series of novel, genetically stable ciliary mutants to address how ciliary beating differs among these cell types. However, several concerns need to be adequately addressed and the manuscript extensively revised before it is suitable for publication.

Major concerns:
CFAP53 (also known as CCDC11) has been reported to cause body laterality defects without the typical respiratory symptoms consistent with a diagnosis for primary ciliary dyskinesia (Narasimhan et al., 2015). In the aforementioned study, anti-human CFAP53 antibody was used to demonstrate localization throughout the entire axoneme of respiratory cilia – the specificity was established by its absence in respiratory cilia from a patient with a homozygous loss-of-function CFAP53 mutation. Here, Ide and co-authors describe mouse Cfap53 localization in pericentriolar satellites in nodal cilia and proximal localization in tracheal cilia. The most convincing data is the localization of Cfap53-Venus at the pericentriolar satellites of nodal cilia, where there is a clear colocalization (yellow overlay) with PCM-1 and gamma tubulin (Fig 3). This Cfap53 localization around the pericentriolar region is appreciated in three separate images (Fig. 3E, 3O, and 3R). However, the localization of CFAP53::Venus in nodal cilia also appears as discrete puncta in the distal ciliary region (see Fig. 3O-Q and Fig. 3R-T) as well as accumulation around the ciliary base. This has significant implications for interpreting the mechanism whereby Cfap53 differentially regulates ODAs by either facilitating its transport into nodal cilia or selectively stabilizing ODAs (position 3 and 8) in the axonemes of tracheal cilia.
The authors also note that Cfap53::Venus localized to the proximal ciliary region in respiratory axonemes (Fig. 3). This interpretation is less convincing and appears to conflict with human CFAP53 data, which shows its native detection along the entire respiratory axoneme (Narasimhan 2015). The localization of Cfap53::Venus using anti-GFP antibody in trachea appears diffuse with more intense signal in the proximal region but also signal in the distal ciliary region. This is in contrast to DNAH11, which shows a clearly defined reactivity in only the proximal ciliary region. The conclusion that Cfap53 is axonemal in tracheal cilia (and possibly nodal cilia) seems sufficient and is consistent with other reports describing CFAP53 in other species, whereas the interpretation that Cfap53 has a proximal localization in mouse trachea requires further clarification as to how / why this would be different. This latter point would be significantly strengthened if the authors can specify whether, in the course of quantifying ODAs in TEMs of Cfap53-mutant cilia, the deficiency of ODAs at positions 3 and 8 (or elsewhere) were in the proximal ciliary region of trachea axonemes as opposed to the distal ciliary region. Furthermore, there is no presentation of native Cfap53 localization by immunofluorescence microscopy, which would be very helpful to corroborate the localization pattern of Cfap53-Venus using anti-GFP antibody. Can the authors comment on why anti-Cfap53 protein (which is included in the material and methods section) is not shown / tested for immunofluorescence microscopy? Have the authors used the published anti-human CFAP53 antibody in Narasimhan 2015 to test cross-reactivity with mouse axonemes?*

Response: We thank this and other reviewers for pointing out this important issue. As suggested by the reviewers, we have re-examined the localization of native Cfap53 protein with the antibody against mouse Cfap53 protein. Unlike Cfap53::Venus protein, native Cfasp53 protein was localized to the entire axoneme of tracheal cilia (now shown in Fig 3M-3R). As requested, we also examined the localization of Cfap53::Venus protein in tracheal cilia of Cfap53::Venus Tg(+), *Cfap53* (-/-) mouse. Cfap53::Venus protein was localized to the tracheal cilia even in the absence of native Cfap53 protein but was absent in the most distal region (S4 Fig). Thus, the absence of Cfap53::Venus protein in the distal-most region of the tracheal cilia is not due to competition with the native protein, but likely due the presence of the Venus tag that possibly prevents efficient transport of the fusion protein to the distal portion. New data on the endogenous Cfap53 protein (obtained with the anti-Cfap53 antibody) are now shown in Fig 3 while localization data on Cfap53::Venus protein have been moved to S4 Fig.

With the new data showing that native Cfap53 protein is localized to the entire axoneme of trachea cilia, it would not be necessary to distinguish the proximal and distal portions of mutant trachea cilia. Nonetheless, we have examined the distal, middle and proximal regions of *Cfap53* (-/-) tracheal cilia by TEM. There was no obvious preferential loss of ODAs either in the proximal or distal region (S8 Fig).

We also examined the localization of native Cfap53 protein in the node cilia with the anti-Cfap53 antibody. As we previously showed for Cfap53::Venus protein, the endogenous Cfap53 protein was mainly localized at the base of node cilia and weakly in their axoneme. This is now shown in Fig 3A-3L.

*Given the points raised above and the interesting result that a subset of ODAs are preferentially affected in tracheal but not nodal cilia, it is conceivable that Cfap53 axonemal localization is restricted to a subset of microtubule doublets (positions 3 and 8) in trachea cilia but targeted to most or all microtubule doublets in nodal cilia. In this alternate model, Cfap53 is axonemal in both tracheal and nodal cilia and predicts that subsets of MTDs are qualitatively different between these cell types. This is not without precedent, as MTDs at position 5 and 6 have a unique connecting structure that is not present among the other MTDs.*

Response: We tried to examine precise localization of Cfap53 protein in the axoneme by immuno-TEM. Unfortunately, however, our antibodies (anti-GFP and anti-Cfap53 antibodies) did not work for immuno-TEM. It is technically demanding (almost impossible) to localize a protein to a specific set of microtubules in vertebrate cilia (in addition to specific immunological signals, one would need to have very clear TEM images showing individual peripheral and central microtubules). The lack of ODAs was preferentially found at position 3 and 8. Although this was statistically significant by circular analysis, the absence of ODAs at positions 3 and 8 was not exclusive, but twice higher than that at other positions. Therefore, we believe that it is more likely that Cfap53 is not localized to specific microtubules, and that positions 3 and 8 lost ODAs preferentially because of the beating motion pattern along that axis.

*Minor Concerns:
References
- Page 11, lines 209-211: the study by McGrath et al., 2003 (reference 29) does not distinguish / define proximal ciliary region in their study whereas the DNAH11 study from the group of Omran (reference 11) is the first to make this distinction. However, both of these studies note localization of Gfp-Dnah11 throughout the entire nodal cilia axoneme. This sentence should be rephrased to more accurately reflect these points.*

Response: We have made the correction – thank you for pointing out this error.

*Phrasing should be modified
- Page 5, lines 63 – 66: “… those with situs inversus and heterotaxy are typically afflicted exclusively with aberrations in laterality…” This suggests that most cases of situs inversus and heterotaxy occur in isolation but this is not entirely accurate as most cases of laterality cases are associated in conjunction with PCD. This should be reworded to convey that there are certain cases / reports of situs inversus / heterotaxy with no accompanying diagnosis of PCD.*

Response: Thank you for the advice. We have reworded this section as suggested.

*The description of Dnah9-/- mice is novel and regarding analysis of laterality, the authors state that 2/2 embryos showed normal situs composition. This is a low number to strictly rule out randomization of laterality and especially heterotaxy – if the authors have not analyzed additional embryos it would be helpful to state that laterality defects especially heterotaxy cannot be fully excluded with the analysis of only 2 embryos. This would be compatible with Dnah9 mRNA protein expression at the node and the possibility that loss of Dnah9 in mice could have very subtle / low frequency effect on laterality development including heterotaxy. Have the authors tested anti-Dnah9 antibody by Western blotting to support its specificity?*

Response: We have examined additional *Dnah9* mutants. Among nine *Dnah9* mutant mice, all showed normal laterality at the birth. Five of them died within 2~3 weeks (at least 4 showed hydrocephalus), while four survived. Among the four live *Dnah9* (-/-) mice, one male was infertile while one female was fertile. When a male *Dnah9* (-/-) mouse was sacrificed at 5 months after the birth, the sperm was morphologically normal and retained normal motility (S11-S12 Videos). This is consistent with the previous observations that DNAH9 is not expressed in human sperm (Whitfield et al., 2019). When two *Dnah9* (-/-) mice were examined for ciliary motility, tracheal cilia and ependymal cilia were motile but exhibited abnormal beating pattern with reduced beating angle and insufficient effective stroke (Fig 6; S7-S10 Videos). Accordingly, *Dnah9* (-/-) mouse showed hydrocephalus as early as a month after birth (4/6 mice examined). These results suggest that Dnah9 is required for normal motility of ependymal and respiratory cilia but is dispensable for the motility of node cilia. As for anti-Dnah9 antibody, the antibody used in this paper (a gift from Dr. Takeda) does not work for western blot. However, this mutant allele (Δex30) is most likely a null allele because exon 30 encodes a motor domain essential for the motility of dynein proteins.

*Regarding the speculation that outer dynein arms in nodal cilia may be composed of a hetero-trimeric complex (page 18, lines 380-383), is there any published evidence to support the existence of three-headed dynein arm complexes in mammalian cilia?*

Response: We have removed the sentence describing a hetero-trimeric complex. There is no evidence for the existence of three-headed dynein in opisthokonta, including mammals. ODAs in node cilia likely possess Dnah5/Dnah11 heterodimer since Dnah9 is absent from node cilia (S6 Fig).

*The title does not appreciably convey the novelty of the findings, as previous publications have already demonstrated a differential role for CFAP53 in tracheal versus nodal ciliary motility. This could be strengthened by possibly mentioning selective versus complete ODA deficiency between these cell types.
As the authors also indicate Cfap53 expression in male testes, can they comment on the fertility status of Cfap53-/- males?*

Response: We have changed the title to “Cfap53 regulates mammalian cilia-type motility patterns through differential localization and recruitment of axonemal dynein components”. *Cfap53*-/- males were infertile. Sperm from *Cfap53-/-* males was morphologically abnormal lacking the tail region (S2 Fig) and were completely immotile (S5 and S6 Video)*.*

*In Figure 9, the authors state that Ttc25 is lost in Cfap53-/- node cilia. However, considering the inset highlighting the cilia, the signal looks much weaker for Ttc25 in heterozygous control (Fig. 9J) than in homozygous null (inset Fig.9M), and cilia are more easily discerned with even some level of colocalization in this sample (Fig. 9O). Can the authors comment further on this?*

Response: We now show cilia at a higher magnification in insets (Fig 9G-9L). The anti-Ttc25 antibody shows a certain amount of background in the cytoplasm. However, since Ttc25 protein is known to localize only to the axoneme, it is quite apparent that Ttc25 is lost from the axonemes of *Cfap53*-/- node cilia.

*Regarding the model of Cfap53 in motile cilia (Fig. 10), point (B) refers to interaction of Cfap53 with IFT components (page 34). Is there published evidence for the interaction of Cfap53 with IFT components?
The authors conclude that Cfap53 interacts with the ODA docking machinery, specifically TTC25, as well as dyneins associated with the outer dynein arms (Dnai1, Dnai2, Dnah11). There are no control lanes for Western blots in Fig. 8C and 8G, which ideally should be compared on the same blot / exposure to interpret strength of signal relative to control. The interaction of Dnah11 fragment aa 1000-1700 with Cfap53 is extremely weak (Fig. 8D) and should be interpreted with caution, as this fragment was more robustly immunoprecipitated than the aa 1-1000 construct (compare band intensities of anti-GFP with Dnah11 1-1000 and Dnah11 1000-1700, top panel) but shows a much weaker / almost negligible signal for Cfap53 (middle panel, anti-HA antibody). Additionally, the expected molecular weight of Ttc25 is approximately 72 kDa, however the blots (Fig. 8E, top and bottom panels) indicate a migration closer to 50 kDa – can the authors provide further information including whether a smaller isoform of Ttc25 was tested?
Regarding the use of NaI solution to discriminate proteins tightly associated with axonemal microtubules, are there published references using this technique to analyze axonemes in other ciliary models / species?
The combined phenotype of Cfap53-/- + Rsph4a-/- mice show a reduction in ODAs in tracheal cilia (Fig. 7M). This is an interesting result but also unexpected as radial spoke proteins are not expected to interact with outer dynein arm proteins. Can the authors further comment on how this synergy may regulate ODAs and also if there are previous studies that support a functional association between radial spoke and outer dynein arms?*

Response: We have repeated the immunoprecipitation experiments and have done western blots with control lanes (revised Fig 8 and S9 Fig). We have adjusted the amount of transfected DNA to normalize the expression level of Dnah11 1-1000 and 1000-1700 aa fragments. We can still detect weak interaction between the 1000-1700 aa fragment and Cfap53 (now shown in revised Fig 8). Dyneins interact with microtubules via their tail region (amino acid residues 1-1367). Although 1000-1700 aa covers a part of the tail region, it lacks the remaining region (1-1000 aa), which may have made the interaction weak. As for the molecular weight of Ttc25 protein, we cloned the mouse Ttc25 sequence (GenBank: AAH50805.2). Mouse Ttc25 protein has 424 amino acids, whereas human TTC25 (GenBank: AAH67297.1) has 665 amino acids. The mouse Ttc25 sequence that we used for our IP experiments probably represents a shorter form of Ttc25.

Regarding the use of Sodium iodide, NaI was used in Wirschell et al., 2013 (https://www.ncbi.nlm.nih.gov/pmc/articles/PMC3818796/) where they extracted DRCs from *Chlamydomonas* axonemes with NaI and NaCl. We now cite this paper in the revised manuscript.

About the combined phenotype of *Cfap53*-/-, *Rsph4a*-/- mice: Radial spokes seem to have different roles in ciliary motility between *Chlamydomonas* and mammals. In *Chlamydomonas*, radial spokes do not interact with the outer arm, but is involved in the pathway that activates ODAs (Oda etal.,2014) (<https://www.ncbi.nlm.nih.gov/pmc/articles/PMC3941055/)>. Thus, flagella of *Chlamydomonas* become immotile in the absence of radial spokes. On the other hand, in mouse and human, lack of radial spokes cause trachea cilia to change their motion pattern (from planar beating to rotation), while node cilia remain unaffected. Therefore radial spokes are required to activate ODAs in *Chlamydomonas*, while they seem to inhibit activation of specific ODAs in mammals. We speculate that in *Cfap53*-/-, *Rsph4a*-/- mice, dyneins at all positions would be activated, forcing tracheal cilia to rotate instead of having the normal planar beating, which would lead to more severe detachment of ODAs from microtubules.

*Cfap53 has been proposed to function as a microtubule-interacting protein within the A-tubule of Chlamydomonas axonemes. If Cfap53 is strictly confined to the inside of the A-tubule, how are interactions with dynein associated components (e.g., Ttc25, Dnah11) possible? Is Cfap53 in some way accessible to the surface of the A-tubule? Also, MIPs have been proposed to confer stability to the 9+0 axoneme, yet the significant phenotype in Cfap53-/- axonemes is partial or complete ODA deficiency (trachea and node, respectively) with no indication of shorter / less stable axonemal MTDs. Can the authors comment on morphology / stability of Cfap53-/- node cilia compared to control? Are their differences between Chlamydomonas and mouse axonemes that can help explain these differences?*

Response: We wanted to examine the precise localization of the Cfap53 protein by immuno-TEM, but unfortunately, our antibodies did not work for this procedure. Therefore, the precise localization of Cfap53 remains unknown. Cfap53 protein may be localized to the surface of the axonemal microtubules and may interact with dynein and DRC. *Cfap53* (-/-) node cilia have normal morphology (Fig 3A-3L, Fig 5A-5F, Fig 9G-9L). We have examined the length of the node cilia (S1 Fig). The length of node cilia was slightly shorter in the *Cfap53* (-/-) embryos, but the length of the tracheal cilia was normal in the mutant. This is now mentioned briefly (page 9).

***Reviewer #3: OVERVIEW:*** *The molecular and structural differences underlying mammalian motile cilia diversity in terms of function and waveform is largely unknown. Here, Ide, Twan and Lu et al have carried out a detailed functional and molecular analysis of the coiled-coil domain containing protein CFAP53 in mammalian motile cilia. Homozygous crispant null alleles of CFAP53 disrupt ciliary beat waveform in the MCCs of the ependymal and respiratory epithelia and show reduced beat frequency in tracheal cells. In contrast, structure and motility of the nodal (9 + 0) cilia is more strongly affected- by TEM that ODAs are heavily depleted. This likely underlies the isolated situs defects, as opposed to more syndromic primary ciliary dyskinesia (PCD), previously published for human patients. Even in the mildly affected MCC axonemes from Cfap53-/- mice, the authors could demonstrate ODAs on specific doublets were preferentially lost, those in the plane of beating in 9 + 2 axonemes. This hypothesis is elegantly test by generating Rsph4a-/- Cfap53+/- - which now show complete loss of ODAs across the field in these rotational mutant MCCs.

The authors have developed an impressive arsenal of BAC transgenic mouse lines to monitor expression and localization of gene of interest CFAP53 as well as two ODA dynein heavy chain tagged lines (DNAH9 and 11). Beyond this study, these reagents are very useful tools for those working in the mammalian motile cilia field. In their BAC transgenics, they show the expression of Cfap53 transcripts in mouse motile ciliated tissues of the mouse and importantly demonstrate that their CFAP53-Venus fusion is functional- rescuing the hydrocephaly phenotype of Cfap53 mutant mice. Using endogenous tagging approaches for axonemal dynein heavy chain fusion lines- they show that nodal but not tracheal cells lack ODA heavy chain staining. Moreover, they suggest that that unlike humans, mice seem to lack DNAH9 from the nodal cilia and do not require it for correct organ positioning (n=2 embryos).

Finally, in fractionation studies, they demonstrate CFAP53 is associated with microtubule axoneme under conditions where ODAs become detached. In a series of transient transfections in HEKs to pulldown interactors of CFAP53- they demonstrate that CFAP53 interacts with selected ODA proteins and the docking complex protein TTC25 but not more distal CCDC114 or 151. They further demonstrate that axonemal localisation of TTC25 is reliant on CFAP53, but not vice versa. This leads to their model which suggests that CFAP53 functions to help transport ODAs into the axoneme and assists in their docking there and that this function is redundant in respiratory cilia where is likely another protein compensates for CFAP53.

Overall, it is a very elegant mouse genetics paper to functionally dissect roles for known human disease gene CFAP53 in diverse mammalian cilia types. The motile-specific phenotypes are unexpected given the KD/morphant phenotypes from other species and mammalian cell culture, where a role in ciliogenesis was reported. However, this story is much cleaner in terms of explaining the phenotypes in patients with CFAP53 variants. Overall, the work is of very high quality, careful analysis and broad significance to the cilia community. Major points are for proper analysis of data presented (quantitation, expanded analysis and/or discussion).

Major points:
1) BAC generation and experiments: While BAC clones are useful for preserving endogenous regulatory elements- levels and patterns of expression- I think these CFAP53 localization experiments were done in the background of wild type CFAP53. Given this, I would caution a little- dampen the emphasis made on differential localizations of CFAP53 between different motile cilia types. ‘Competition’ between tagged and untagged isoforms for cilia localization could be limited- we know this is the case for IFT proteins. You get higher labelling density within cilia if the tagged-isoform is the only one expressed. One workaround would be to validate using the endogenous CFAP53 expression using the antibody they have generated- for Fig8 if it works by IF.
i. Regardless, with the current images, quantitative image analysis of the distributions would be very helpful- average arbitrary units of fluorescence down the axonemes of different cilia types.*

**Response:** We thank this and other reviewers for pointing out this important issue. As mentioned above, we have re-examined the localization of native Cfap53 protein with an antibody against mouse Cfap53 protein. Unlike Cfap53::Venus protein, endogenous Cfap53 protein was localized to the entire axoneme of trachea cilial (now shown in Fig 3M-3R). As requested, we also examined the localization of Cfap53::Venus protein in trachea cilia of Cfap53::Venus Tg(+), *Cfap53* (-/-) mouse. Cfap53::Venus protein was localized to the trachea cilia even in the absence of native Cfap53 protein but was absent in the most distal region (S4 Fig). The absence of Cfap53::Venus protein in the distal most region of the tracheal cilia, is not due to competition with the native protein but likely due to the presence of the Venus tag that might have prevented the efficient transport of the fusion protein to the distal portion. New data on the endogenous Cfap53 protein (obtained with the anti-Cfap53 antibody) are now shown in Fig 3 while localization data on Cfap53::Venus protein are moved to S4 Fig. We also examined the localization of native Cfap53 protein in the node cilia with the anti-Cfap53 antibody. Native Cfap53 protein was mainly localized at the base of node cilia and weakly in the axoneme (as Cfap53::Venus protein). This is now shown in Fig 3. For Dnah9 that is preferentially localized to the distal region of trachea cilia, arbitrary units of fluorescence are shown for the proximal and distal regions (S7 Fig).

*ii. Also, I did not find the numbers of BAC integrations (often concatomers, as well as locus effects) expression relative to endogenous locus. If the authors have this information- authors should include in the methods how many founders, how many copies, relative expression, screening?*

Response: We have examined the copy number of integrated BAC transgenes by quantitative PCR with various genomic DNAs. With the control DNA from Dnah11::Venus knock-in mouse (Dnah11::Venus/Venus), the copy number of *Venus* in two lines of Cfap53::Venus transgenic mouse was estimated to be 1 copy or 2 copies per cell (S3 Fig). Usually, the copy number of a BAC transgene is low because it is large.

*iii. The literature suggests that CFAP53 is widely expressed in primary ciliated cells where it seems to function in ciliogenesis (i.e. Silva et al 2015) - however none of the previous work in human cell lines RPE1 or Xenopus over-expression shows cilia localization. This is an important finding of the current study and discrepancy should be discussed a little more clearly. This is important for the field.*

Response: Our current work with the mouse (Fig 1) and our previous work with the zebrafish (Narasimhan et al. 2015) are consistent and indicate that Cfap53 is expressed in cells with motile cilia but absent in cells with immotile primary cilia. Moreover, knockdown in zebrafish and knockout in the mouse has shown that Cfap53 has a function in motile cilia and not primary cilia. We now discussed this briefly in the discussion (page 16).

*2) CFAP53 allele characterization: Minor point for two CRIPSR alleles- how do the genomic changes affect protein (i.e. premature termination, in frame deletion). Please add in this information to Fig.2 A. Were the authors able to validate these predictions with their endogenous antibody they use in Figure 8A? Is any protein still present- truncated proteins are not null alleles. This is important given the potential discrepancies with the morphant/KD experiments in Xenopus and zebrafish- Mitchell and Mahjoub, as well as Khoka labs, as well as mammalian cells where ciliogenesis is affected. This should be discussed.*

Response: Two mutant alleles are most likely null alleles. First, deletion of these exons can result only in the production of only a highly truncated protein. Secondly, the Cfap53 antibody, which was raised against the C-terminal region of Cfap53, failed to detect native Cfap53 protein in tracheal cilia of *Cfap53* (-/-) mouse (Fig. 3P-3R).

*3) DNAH9-Venus and DNAH11-Venus localizations: Beautiful endogenous tagging approaches for axonemal dynein heavy chain fusion lines will be great resources for the community. If this paper is to be where they are referenced/introduced, I suggest however that more image analysis is needed in terms of the proximal-distal intensities- could a plot of average axoneme distributions for Venus-tagged molecules be shown next to the image panels in Figure 5 and 6. For example, DNAH11 is expected to be proximal in its localization but in fact in 5G and 5J it looks like it is excluded (non-overlapping) with the AcTub staining. A graph capturing the signals would be much easier to interpret. Similarly, Fig6, DNAH9-Venus looks much more broadly than restricted to the distal tip. Moreover, do these boundaries change at all in the mutants? In the text authors make the statement (lines 218)- ‘distal localization of Dnah9 was maintained, although its level was reduced compared with that apparent in control mice’. There is no quantitation here- quantitating the signals similar to distribution is required if these statements are to be made. For example, 6J-L- if you averaged the DNAH11/acTub signal intensity, the apparent decreased in DNAH11 would likely not hold. Again quantitation for the nodal cilia would also be helpful- magnifications of the DNAH9-Venus nodal cilia would also be necessary.*

Response: We have re-examined proximal/distal localization of Dnah11 and Dnah9 protein in tracheal cilia. We now provide a new fluorescence image of Dnah11::Venus (S5C Fig) showing its proximal localization. We also provide a new immunostaining image of Dnah9::Venus protein showing its distal localization (S7C Fig). We also show quantitative analysis of Dnah9 signal intensity (S7C Fig), which further confirms distal localization of Dnah9 in trachea cilia.

*Minor points:
1) Phenotype: CFAP53 is highly expressed in testes- is there a sperm defect suggest it is a trafficking module for the paralogues DNAH17 and DNAH8? Or is it not required? Similarly, given the hydrocephaly phenotype why not characterize ependymal distribution (localization and levels) of the heavy chains in these cilia, or TEM analysis? By only looking at two cilia types, it is possible that the respiratory cilia are the odd ones out and not the nodal cilia (i.e. ependymal cilia are also affected more severely or sperm motility). How penetrant is the gross hydrocephaly phenotype- 100% have domed heads or subclinical between animals? Along the same vein, which background were the animals made on and maintained on to understand this phenotype.*

Response: We have examined fertility of *Cfap53* KO males and motility of their sperm. *Cfap53* KO males were infertile. Their sperm were morphologically abnormal lacking the tail region (S2 Fig) and were completely immotile (S5 and S6 Video). We were unable to examine Dnah17 and Dnah8 because mutant sperm was morphologically very abnormal. Hydrocephalus phenotype was highly penetrant: all the *Cfap53* mutant mice (14/14) exhibited hydrocephalus before they died around 4~6 weeks after the birth. Motility of ependymal cilia from *Cfap53* mutant mice is affected in a pattern similar to the tracheal cilia (Fig. 2G). *Cfap53* KO mouse was generated and maintained in the C57Black background. Cfap53::Venus BAC transgenic mouse was generated with the BDF1 mice. This is now mentioned in the Materials and Methods section (page 21-22).

*2) Nomenclature: Throughout mouse proteins should be in all caps (i.e. CFAP53) whereas genes italics title case (i.e. Cfap53). This needs careful and extensive changing throughout the manuscript- TTC25, DNAH9, DNAH11 and intermediate chains please use DNAI1 and DNAI2, which is the correct form of the protein.*

Response: While human proteins/genes are all capitalized (as CFAP53), mouse proteins/genes are commonly described with the first letter capitalized (such as Cfap53) (please see: http://www.informatics.jax.org).

*3) Figure 2H- the spread in the control frequencies is unusual- hyperkinetic to hypokinetic almost overlapping mutants- but it is a ‘pool’ of control animals-wild type and het. We recommend identifying genotypes by symbols- are the hets intermediate?*

Response: We have analyzed beating frequency again for wild-type, hets and homozygous mutants, and have replaced Fig. 2H. Homozygous mutants showed reduced beating frequency, which was statistically significant.

*4) Along with major point 3, the language is currently strong in suggesting that that unlike humans, mice seem to lack DNAH9 from the nodal cilia and do not require it for correct organ positioning (n=2 embryos). Given the small numbers of Dnah9 mutants examined, I would temper conclusions to ‘suggesting DNAH9 is not expressed in, nor required for mouse nodal cilia motility’. Given mRNA expression by WISH, can the authors rule out issues with the signal-noise ratio for low protein expression may confound this interpretation for DNAH9? The DNAH11-Venus levels (GFP IF) are also significantly lower than say DNAH5 IF- the enrichment in the cilia not very apparent (see Figure 5A- cytoplasm to axoneme). A GFP-IP of E8.5 embryos pooled- no tagged protein pulled down? Something else to bolster. If not use language, like ‘appears’ and ‘suggests’.*

Response: As for the presence or absence of Dnah9 protein in the mouse node, we now mention that “we failed to detect Dnah9 protein in node cilia” (page 12). We have examined additional *Dnah9* mutants. Among nine *Dnah9* mutant mice, all showed normal laterality at the birth (Figure 6). Six of them died within 2~3 weeks (at least 4 showed hydrocephalus), while three survived. Among the three live *Dnah9* (-/-) mice, one male was infertile while one female was fertile. When a male *Dnah9* (-/-) mouse was sacrificed at 2 months after the birth, the sperm was motile without apparent anomalies, while trachea cilia ependymal cilia showed reduced beating angle making the effective stroke insufficient. These results suggest that Dnah9 is required for the normal beat pattern of trachea and brain cilia but is dispensable for the motility of node cilia.

*5) Inner dynein arms: While the authors have demonstrated that there is an effect on ODAs in Cfap53 mutants, there is no mention of the effect on IDAs. While this is not necessarily expected given the evidence from patient cells it is nevertheless an important consideration. What would the mechanism be? This could be mitigated with expanded discussion on previous observations about IDAs and why you think it is right not to investigate them in this study. Quantitate IDAs from mutant TEM*

Response: Unfortunately, it is extremely difficult to detect IDAs in TEM images of vertebrate motile cilia, making it impossible to quantitate the number of IDAs even in WT tracheal cilia (in fact, there is no paper that reports quantitative defects of IDA in vertebrate mutants). Furthermore, there are no good antibodies for IDA components. We now discuss this briefly (page 18).

*6) Clarify Figure 9 shows some images of the node, however the cilia are very hard to see with the staining used. The labelling of G-I is also a bit unclear. Label G-I to indicate that these are Cfap53+/+ mice. Make the magnified boxes larger and make the entire image brighter so the cilia are easier to see.*

Response: We have improved Figure 9 by making the magnified boxes larger. It is now clear that Ttc25 protein is absent in the axoneme of *Cfap53* (-/-) node cilia.

*7) Line 33-34: edit to ‘albeit with an altered beat pattern’*

Response: We have corrected this.

*8) Centriolar satellites is used as plural by the field, even in cells with a single cilium like the node. They are highly dynamic and tend to migrate, fuse and divide in short time scales. See line 34 and throughout.*

Response: Thank you for pointing this out, and we now use centriolar satellites as plural throughout the text.

*9) Lines 46-50: This sentence is too long. Split into 9+0 and 9+2 motile cilia sentences, each with a full stop.*

Response: We have amended this.

*10) Line 149: typo ‘lacking exon 2’.*

Response: We have amended this.

*11) Line 171: typo ‘that the Cfap53.’*

Response: We have amended this.

*12) Line 267: for clarity add ‘TT25 is thought to be most proximal to the microtubule’ currently unclear proximal to what.*

Response: We meant that Ttc25 is most proximal to the axonemal microtubules, relative to other docking complex members such as Ccdc114, Ccdc151 etc. We have clarified this in the text.

*13) Lines 355-359: The sentence needs work ‘It may also not necessarily be the issue of absence of a particular protein from 9+0 and presence of it in 9+2 cilia that confers the observed differences on the effects of the loss of CFAP53 on ODAs and DC in the two types of cilia, rather like CFAP53, a protein present in both cilia-types but with differential localization and function in each kind of cilia could also fulfil this role.’ Wouldn’t a differential localization and function likely involve the presence/absence of another protein to execute these changes- the logic is fuzzy.*

Response: We have amended this.

*14) Line 381-383: Isn’t it much more likely that there is a heterogenous mix heterodimers than a heterotrimer. i.e. not spatially separated dimers in the human node.*

Response: We have corrected the sentence as suggested by the reviewer.

*15) Line 448: Specify what is the ‘blocking solution’.*

Response: Blocking solution is the blocking reagent Perkin Elmer FP1020. This is now mentioned in Material and Methods (page 23).

*16) Line 451: Change to ‘Can Get SolutionTM A/B’, and on line 466.*

Response: We have amended this.

*17) Line 637: Change to ‘Figure Legends’*

Response: We have amended this.

*18) Line 704: typo ‘ODAs are…’*

Response: We have amended this.

*19) Line 754: reference for the interaction with IFTs if shown, if presumed state explicitly.*

Response: We have amended this.

*20) Line 781: provide n= for how many animals were examined for laterality defects in the legend.*

Response: The number of animals examined has been indicated in S5B Fig.

*21) Accessibility: most of the IF data is two color/merge- consider choosing red-green colour-blind friendly combinations.* [*https://imagej.nih.gov/ij/docs/guide/146-9.html*](https://imagej.nih.gov/ij/docs/guide/146-9.html) *and* [*https://www.ascb.org/science-news/how-to-make-scientific-figures-accessible-to-readers-with-color-blindness/*](https://www.ascb.org/science-news/how-to-make-scientific-figures-accessible-to-readers-with-color-blindness/)

Response: New data (Fig 3, Fig 9, S4 Fig, S5 Fig, S7 Fig) are now shown in color-blind friendly combination (green-magenta).
